# Short-Term Effects of Cold Therapy and Kinesio Taping on Pain Relief and Upper Extremity Functionality in Individuals with Rotator Cuff Tendonitis: A Randomized Study

**DOI:** 10.3390/medicina60081188

**Published:** 2024-07-23

**Authors:** Elif Durgut, Hulya Nilgun Gurses, Kerem Bilsel, Kubra Alpay, Zeynep Hosbay, Gokcer Uzer, Fatih Yıldız, Nurzat Elmalı

**Affiliations:** 1 Department of Physiotherapy and Rehabilitation, Faculty of Health Sciences, Bezmialem Vakif University, 34050 Istanbul, Turkey; edurgut@bezmialem.edu.tr (E.D.); kalpay@bezmialem.edu.tr (K.A.); 2 Department of Orthopaedics and Traumatology, Faculty of Medicine, Acıbadem Mehmet Ali Aydınlar University, 34752 Istanbul, Turkey; kerem.bilsel@acibadem.com; 3 Department of Physiotherapy and Rehabilitation, Faculty of Health Sciences, Biruni University, 34015 Istanbul, Turkey; zhosbay@biruni.edu.tr; 4 Department of Orthopaedics and Traumatology, Faculty of Medicine, Bezmialem Vakif University, 34093 Istanbul, Turkey; guzer@bezmialem.edu.tr (G.U.); fyildiz@bezmialem.edu.tr (F.Y.); nelmali@bezmialem.edu.tr (N.E.)

**Keywords:** rotator cuff tendonitis, Kinesio taping, cold, pain, function

## Abstract

*Background and Objectives*: Rotator cuff tendonitis (RCT) is one of the most common shoulder pathologies. It causes pain, limits shoulder joint movements, and impairs function. Despite various treatment methods, there are currently no specific guidelines regarding the most effective intervention for RCT. To the best of our knowledge, no studies have compared the effects of Kinesio taping (KT) and cold therapy (CT) on individuals with RCT. To this end, this study aimed to investigate and compare the short-term effects of KT and CT on pain relief and upper extremity functionality in individuals with RCT. *Materials and Methods*: One hundred and fourteen individuals were assessed for eligibility. Fifty-two individuals with RCT who met the inclusion criteria and agreed to participate were randomly allocated into either the KT or the CT group. A standardized home exercise program was given to all the participants. Their pain intensity, upper extremity function, shoulder range of motion (ROM), and grip strength were evaluated initially and after the three days of KT or CT applications. *Results*: All the assessment values significantly improved in the KT group. In the CT group, only the pain scores (except for the numerical rating scale (NRS) pain score during activity) were significantly improved in the CT group at the end of the third day of application compared to the initial values (*p* < 0.05). For all the measurement outcomes, the effects of time × group interactions were statistically significant (*p* < 0.05) in favor of the KT group, except for the resting pain (*p* = 0.688). *Conclusions*: The findings suggest that KT and CT could be used as adjunctive modalities to exercise for resting and night pain relief in patients with RCT. KT also had positive effects on the activity pain, function, ROM, and grip strength. The use of KT along with an exercise program could be a more effective therapeutic choice than the use of CT for improving night pain, activity pain, and upper extremity function during the short-term rehabilitation of RCT patients.

## 1. Introduction

Rotator cuff tendonitis (RCT) is one of the most common shoulder pathologies, causing pain, limited shoulder joint movements, and impaired function [1]. Some extrinsic and intrinsic factors, such as overuse activities, trauma, and degenerative changes in the shoulder joint, or a combination of these factors, are associated with the development of RCT. Inflammation of the tendons can cause swelling; irritation; discomfort; pain, especially during activity; limited shoulder movement; muscular weakness; and ultimately, functional disability. RCT can lead to degenerative changes or the partial or total rupture of the tendon in the later stages if left untreated [1,2]. All of these considerations highlight the need for effective RCT interventions to avoid worsening the inflammation that leads to degeneration and negatively affects upper extremity function and the activities of daily living [3].

Patient education; relative rest; medical treatments, such as the use of analgesics and non-steroidal anti-inflammatory drugs (NSAIDs); corticosteroid injections; physiotherapy rehabilitation approaches, especially laser therapy; ultrasound; transcutaneous electrical nerve stimulation (TENS); exercise training; and cold therapy (CT) (also known as cryotherapy) are the most common treatment options applied to alleviate the symptoms of RCT [3,4,5]. Cold application, which dates back to ancient times as a therapeutic agent [6], is still a widely preferred intervention in soft tissue therapy for pain, edema, and inflammation management. CT is used for its analgesic, anti-inflammatory, vasoconstrictive, and antioxidative effects in shoulder pathologies by reducing inflammation, oxidative stress, temperature, nerve conduction velocity, swelling, enzymatic activity, blood circulation, and the transmission of pain signals [3,4,5,6]. It is also preferred for improving shoulder function [3]. However, its results depend on the technique used, the duration of application, the reduction in the skin temperature, and the depth of cold penetration. Among CT techniques, an ice pack is a low-cost device that is easily accessible [7]. It leads to a decrease in the tissue temperature and induces physiological changes to a depth of at least one centimeter [8]. Kinesio taping (KT) is one of the most popular methods and is a commonly used rehabilitation modality in clinical practice to rehabilitate musculoskeletal disorders. KT causes physiological changes in the skin, circulation, fascia, muscles, and joints. It lifts the skin, reduces the pressure on tissues, and improves the lymphatic fluid, increasing blood circulation, reducing edema, preventing the stimulation of pain receptors, and accelerating tissue healing. It also provides support over the joint and a positional stimulus through the skin, improving postural alignment and functional movement [9,10,11,12]. It has recently become a preferred option for pain management and functional activity improvement in the treatment of RCT [13,14,15]. Despite these various treatment methods, there are currently no specific guidelines regarding the most appropriate and effective intervention for RCT treatment. This is mainly because adequate, high-quality studies on RCT management are lacking, and more randomized controlled trials are needed to investigate the superiority of the treatment options used [5]. 

This prospective and randomized study aimed to investigate and compare the short-term effects of KT and CT on pain relief and the upper extremity functionality in individuals with RCT. For the current study, our hypotheses are as follows:

(1). Both CT and KT applications, in conjunction with an exercise program, would improve pain and function in the short-term rehabilitation of RCT;
(2). CT would be more effective than KT in improving pain and upper extremity function in the short term in individuals with RCT;
(3). KT application, compared with CT, would be more effective for improving pain and upper extremity function in the short term in individuals with RCT.

## 2. Materials and Methods

### 2.1. Study Design and Protocol

A double-blinded, prospective, and randomized study was conducted between June 2021 and February 2023. One hundred and fourteen individuals diagnosed with RCT were assessed for eligibility, and fifty-two participants aged between 30 and 60 years were selected and referred from the Bezmialem Vakif University Department of Orthopedics and Traumatology to the Department of Physiotherapy and Rehabilitation. Forty-six individuals diagnosed with RCT by experienced orthopedic surgeons were included in the study. The selection criteria for the participants were as follows: having a diagnosis of RCT with the exclusion of other shoulder pathologies using magnetic resonance imaging (MRI) and specific tests, including the Neer test, Speed test, O’Brien test, and Jobe test, and having rotator cuff edema. The exclusion criteria were as follows: (1) a glenohumeral joint dislocation/subluxation; (2) an acromioclavicular sprain; (3) a rotator cuff tear; (4) glenohumeral joint instability; (5) calcific tendinitis of the shoulder; (6) acromioclavicular joint pathologies; (7) hyperlaxity; (8) any fracture in the shoulder; (9) diabetes, thyroid disease, or any vascular or rheumatologic disease; (10) glenohumeral joint deformities; (11) a superior labrum anteroposterior (SLAP) lesion; (12) shoulder pain lasting more than six months; (13) a history of shoulder surgery; and (14) intra-articular steroid injections.

After the initial assessment, the participants were randomly allocated to either the KT or the CT group using a numbered series of 52 prefilled envelopes specifying the group assignments, which were generated with a computer-based program by an independent researcher who was not involved in the recruitment, interventions, or assessments. The participants were unaware of their group assignments. The assessments and interventions were applied by different physiotherapists. Physiotherapists who were unaware of the intervention protocols and the assigned groups performed the assessment procedures. Both interventions were performed by different physiotherapists, and the same intervention was carried out by the same physiotherapist each time. During the study, the participants were asked not to take palliative and/or anti-inflammatory drugs or participate in a different intervention program. All the participants were assessed after receiving three days of the KT and CT applications from the same physiotherapist.

This study was approved by the ethics committee of a university hospital (protocol number: 71306642-050.01.04) and registered with the ClinicalTrials.gov website (registration number: NCT06425913). The study was conducted in accordance with the Declaration of Helsinki. Written informed consent was obtained from each participant.

### 2.2. Interventions

The KT and CT groups were also included in a standardized home exercise program, including pain-free shoulder ROM exercises, isometric exercises, and stretching exercises for the posterior capsule. After the first assessments, the physiotherapists performing the interventions taught the exercise program to the participants until they were able to perform the exercise accurately on their own. All the participants were instructed to perform each exercise three times daily for three days with ten repetitions.

#### 2.2.1. Kinesio-Taping Application

**Figure 1:**
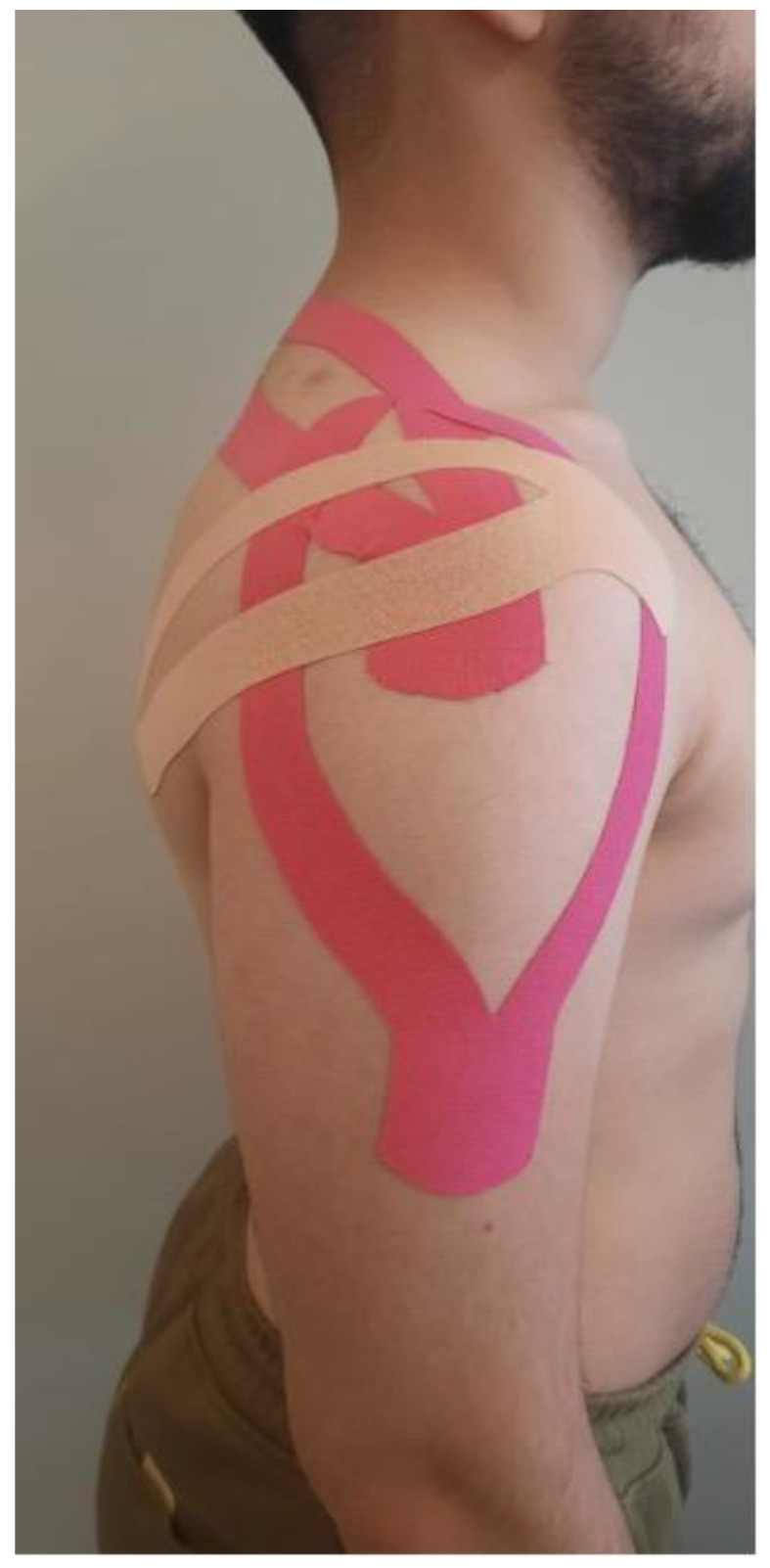
Kinesio-taping method.

Before the KT application, the skin was shaved and cleaned with alcohol to eliminate any oil, lotion, or moisture that could limit the adhesive’s ability to adhere. KT was applied to the affected shoulder after an initial assessment by a certificated physiotherapist with over ten years of experience in Kinesio taping. After three days, the participants were re-evaluated. The KT application was made according to the protocol for rotator cuff impingement or tendonitis, as suggested by Kase et al. [16]. This KT method involved three techniques (Figure 1):

1. The application of supraspinatus muscle taping from the insertion to the origin (inhibition technique—Y strip): Firstly, the length of the Y strip was determined by measuring from the acromion to the spine scapula. Then, with the patient in a sitting position, the base of the Kinesio Y strip was placed 2 inches below the greater tuberosity of the humerus, with no tension. The subject then moved into shoulder adduction behind the back, with lateral neck flexion to the opposite side. Paper-off tension was applied to the Kinesio Y strip. The superior tail was applied superiorly to the spinous process of the scapula, between the upper and middle trapezius muscles, and ended at the supraspinatus fossa on the superior medial border of the scapula. The inferior tail was applied along the spinous process of the scapula. The distal 1–2 inches of the KT Y strip were applied with no tension. 

2. The application of deltoid muscle taping from the insertion to the origin (inhibition technique—Y strip): First, the length of the strip was determined by measuring from the acromion process to the deltoid tuberosity. With the patient in a sitting position, the base of the Kinesio Y strip was placed 2 inches below the deltoid tuberosity of the humerus with no tension. Both the anterior and posterior tails were applied with paper-off tension. The shoulder was placed in abduction to 90%, with external rotation and horizontal extension. The anterior tail was applied along the outer border of the anterior deltoid to approximately the AC joint. The last 2 inches were applied without tension. Then, the subject moved their shoulder into horizontal flexion with internal rotation while maintaining abduction. The posterior tail was applied along the outer border of the posterior deltoid to approximately the acromioclavicular joint. The last 2 inches of the strip were applied without tension.

3. The application of the mechanical correction technique with tension on the base: The base of a 6–8-inch-long Kinesio Y strip was placed on the anterior aspect of the shoulder in the area of the coracoid process, the most painful region, with no tension. One of the physiotherapist’s hands held the base to ensure that no tension was added. Moderate to severe tension (50–75% of the maximum tension) was applied with inward pressure surrounding the area of pain over approximately half of the Kinesio Y strip’s length. When approximately half of the Kinesio Y strip was applied, the physiotherapist slid the hand that was holding the base up to the point of the end tension on the Kinesio Tex^®^Tape (Albuquerque, NM, USA). The physiotherapist then had the subject move into shoulder flexion with horizontal flexion. The tails of the Kinesio Y strip were applied in a splayed-out pattern to dissipate the created force; no tension was applied to the tails.

At the end of all the techniques, the physiotherapist initiated the glue activation prior to any shoulder movement. If the participant developed any type of skin reaction or felt uncomfortable, they were instructed to remove the tape. 

#### 2.2.2. Cold Application

Ice packs were utilized for the cold application. The initial application was administered by the physiotherapist. In a sitting position, a pack was wrapped in a thin towel and placed on the affected shoulder joint, including the painful locations. During the application, the participant was closely observed for discomfort or adverse reactions (redness, burning, numbness, itching, etc.). The cold application was continued for 20 min. After the first application, the participants were instructed to apply ice for 20 min five times a day for three days at home or work. To ensure that the participants were able to follow the program properly, they were instructed to keep a diary of their cold applications.

### 2.3. Outcome Measures

During the initial and final assessments (after three days of the applications), the pain intensity of the subjects was evaluated with the numerical rating scale (NRS); the upper extremity functionality was evaluated with the disability of arm, shoulder, and hand (DASH) questionnaire and the shoulder pain and disability index (SPADI); the range of motion was assessed using a goniometer; and the grip strength was evaluated with a dynamometer.

Numerical rating scale (NRS): The pain severity was assessed using the NRS, for which each subject was asked to rate his/her perceived pain. An 11-point NRS was used from 0 to 10, where 0 means no pain and 10 means the worst possible pain. This scale was scored during the night, at rest, and during activity. The NRS, which is the most commonly used scale to assess pain intensity in clinical practice, is a reliable and valid tool for performing pain assessments in subjects with musculoskeletal impairments of the limbs [17]. 

Disability of the arm, shoulder, and hand (DASH) questionnaire: DASH is a self-reported questionnaire designed for evaluating the functional level of the upper extremities. It is a 30-item scale that addresses the difficulty in performing various physical activities that require upper extremity function (physical function, 21 items); the symptoms of pain, activity-related pain, tingling, weakness, or stiffness (pain symptoms, 5 items); and the impact of disability and symptoms on social activities, work, sleep, and psychological well-being (emotional and social function, 4 items). Each item is scored between 1 and 5. A score of 1 indicates no strain, and a score of 5 indicates an inability to perform the specified activity. The DASH total score ranges from 0 to 100 (0 points = no disability and 100 points = most severe disability). Lower scores indicate a better functional level [18].

Shoulder pain and disability index (SPADI): The SPADI is a self-administered questionnaire developed to measure the pain and disability associated with shoulder pathologies in people with shoulder pain of a musculoskeletal, neurogenic, or undetermined origin. It consists of 13 items that assess two domains: a 5-item subscale that measures pain and an 8-item subscale that measures disability. The items of both domains are scored on a numerical rating scale ranging from 0 to 10, where 0 = no pain/no disability and 10 = worst pain imaginable/so difficult that help was required. Each domain score is summed and transformed to a total score out of 100. The maximum score (100 points) indicates the highest degree of disability [18,19].

Range of motion (ROM): The active range of motion (ROM) of the affected shoulder, including flexion, abduction, external rotation, and internal rotation, was assessed using a universal goniometer by following the protocol reported by the American Academy of Orthopaedic Surgeons (AAOS) [20]. The shoulder ROM measurements were performed while the participants were lying supine. Shoulder flexion was evaluated in the sagittal plane with the arm at the side and the hand pronated, while shoulder abduction was assessed in the frontal plane with the arm at the side and the shoulder externally rotated to achieve maximum abduction. The external and internal rotation of the shoulder were measured in the transverse plane with the arm abducted to 90°, the elbow flexed to 90°, the hand pronated, and the forearm perpendicular to the floor. In total, three measurements were carried out for flexion, abduction, external rotation, and internal rotation, and the average/mean value was calculated. All the goniometer measurements were recorded in the same order for all the participants to prevent inconsistencies in the measurement protocol [21]. 

Grip strength: A Jamar^®^ hydraulic hand dynamometer (Irvington, NY, USA) was used to assess the hand grip strength of the affected side. All the measurements were performed as recommended by the American Society for Hand Therapists, with the participant seated, their shoulder in adduction, the elbow flexed at 90°, and the forearm in a neutral position. All the tests were repeated three times, and the mean value was recorded as the kilogram-force [22].

### 2.4. Data Analysis and Sample Size

The data analysis was performed using SPSS version 20 (SPSS Inc., Chicago, IL, USA). The Shapiro–Wilk and Levene tests were used to determine normality and homogeneity. A between-group analysis of the categorical data and initial variables was performed using the Chi-squared test and an independent sample *t*-test. Moreover, 95% confidence intervals were used for initial data comparisons between groups. Within-group comparisons were performed using a paired sample *t*-test. A mixed-model analysis of variance (time × group interaction) was performed to compare the groups. Partial-eta squared (η^2^) was considered the effect size. The significance level was accepted as *p* < 0.05. The outcomes were expressed as the mean and standard deviation, and categorical data were reported as numbers (n) and percentages (%). 

The G*Power 3.1 software was used to estimate the sample size with α = 0.05 and a power of [1 − β] = 0.80 [23]. Given a mean difference of 2.05 and a standard deviation of 2.30 in the NRS between groups [24], the required sample size was estimated as 21 participants per group. Considering the potential for drop-out during the study, the sample size was increased by 20%, and 26 participants per group were included. Post hoc power analysis revealed that with the current sample size of 23 per group, a mean difference of 1.91 and a standard deviation of 1.95 in the NRS activity pain score, the study had 90% power at α = 0.05.

## 3. Results

**Figure 2:**
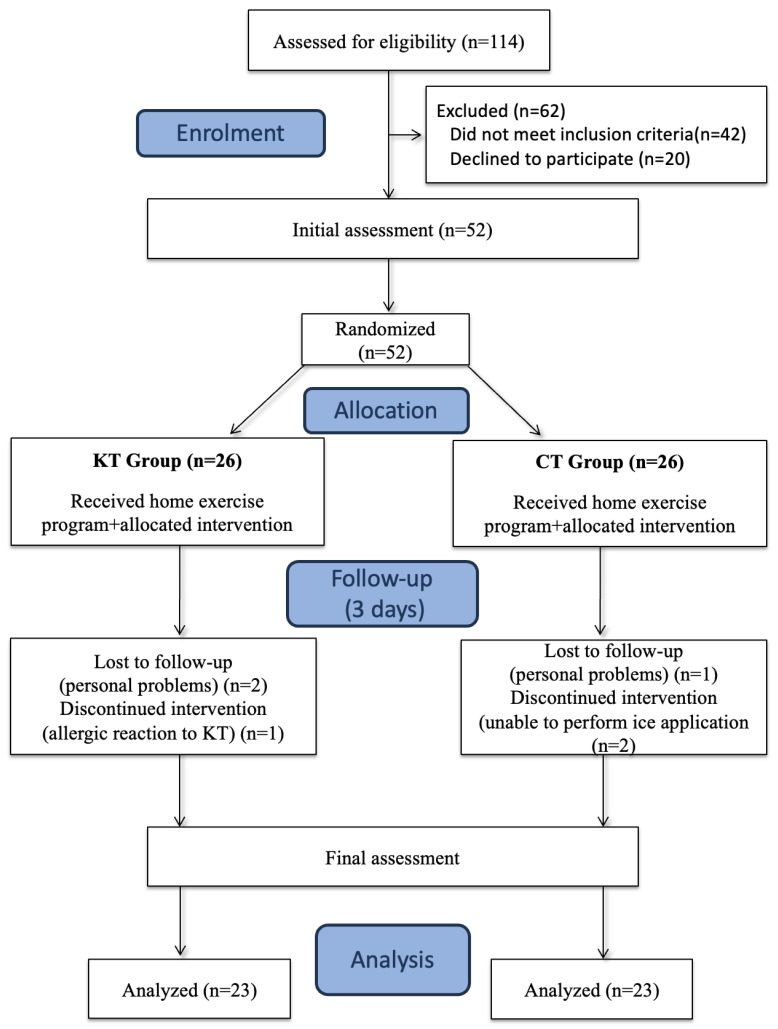
Study flow chart.

One hundred and fourteen individuals with RCT who had been admitted to the Department of Orthopaedics and Traumatology of a university hospital were assessed for eligibility; a total of sixty-two individuals were excluded for not meeting the inclusion criteria or because they declined to participate. Fifty-two participants were randomized into two groups. A total of 26 participants for each group were included in the study, and a total of 46 participants completed the study, with 6 drop-outs (Figure 2). 

The demographic and initial assessments of the groups are presented in Table 1. There were no significant differences in these parameters between the groups (*p* > 0.05).

The effects of the interventions on the NRS, DASH, and SPADI scores, as well as the ROM and grip strength values, are shown in Table 2. The pain scores according to the NRS and SPADI significantly improved in both groups (*p* < 0.05), except for the activity pain score of the NRS in the CT group (*p* = 0.829). The KT group also experienced substantial improvements in the DASH, SPADI—disability, and SPADI—total scores, as well as all the ROM and grip strength values (*p* ≤ 0.001), whereas no significant changes were observed in any of these variables in the CT group (*p* > 0.05). Box plots of changes from initial to final in NRS, SPADI, and DASH questionnaire scores and shoulder ROM and hand grip strength values of KT and CT groups are given in the Supplementary Materials (Figures S1–S4). 

The results of a mixed-model analysis of variance indicated that the time × group interactions were significant (*p* < 0.05) for all the measurement outcomes except the resting pain (*p* = 0.688) (Table 2). The KT group showed significant improvements in pain relief (except for the resting pain), function, the ROM, and the grip strength compared with the CT group.

## 4. Discussion

This study aimed to investigate and compare the short-term effects of KT and CT on pain relief and upper extremity function in individuals with RCT. KT improved pain relief, function, the shoulder ROM, and the hand grip strength, while CT ameliorated only the pain (except the activity pain) in the short term. However, KT was found to be superior in this study in terms of pain relief (except for pain during rest), function, the ROM, and the grip strength compared to CT.

Pain, an important clinical symptom of RCT, limits movement, reduces physical function, and makes it difficult for individuals to perform daily activities over time. Individuals with RCT have pain, especially during activity, that leads to functional limitations. Some patients also experience pain at night. Studies have emphasized that pain, the most frequently reported symptom by patients with RCT, is generally associated with a restriction in the shoulder ROM and muscle weakness, as well as impeded activities of daily living [2,25,26]. Therefore, controlling the pain at the earliest stage is essential to reduce the risk of worsening RCT (the partial or complete tear of tendons, rotator cuff dysfunction, etc.) and enhance the function [27]. 

In this study, the pain scores according to the NRS and SPADI decreased significantly compared to the pre-treatment values in both groups, except for the pain as scored by the NRS during activity in the CT group. CT, the safe, simple, and oldest intervention, is used for acute or chronic pain control as part of a multimodal pain management approach [28,29]. In addition, CT is often recommended for shoulder pain [30,31]. It has been shown to alleviate pain in shoulder impingement syndrome [32], pre-postoperative shoulder surgery [33,34], and rotator cuff tendinopathy [3]. The mechanism underlying its analgesic effect is thought to occur through reducing inflammation, edema, oxidative stress, concentrations of inflammatory mediators that irritate peripheral nociceptors, the nerve conduction velocity in pain fibers, and pain sensations; aid in the healing process of muscle damage; delay the onset of an inflammatory response; and inhibit the transmission of pain signals to the dorsal horn of the spinal cord. Cold applications may also act as a counterirritant by stimulating central pain pathways, which then activate descending inhibitory pathways to block pain transmission to the brain [28,35]. Regarding the exact mechanism behind the beneficial effects of CT in tendons, Zhang et al. [36] conducted a study on mice. The authors stated that CT’s ability to reduce pain for tendon injuries may be attributable to its ability to decrease COX-2 (cyclooxygenase-2) protein expression and prostaglandin E2 production in tendons. Prostaglandin E2 is a highly active inflammatory molecule that causes pain and induces vasodilatation and hyperalgesia. Although CT is a popular treatment to relieve pain and is justified as a safe alternative to treating injured tendons, there is limited knowledge about this treatment in the literature. An evidence-based protocol for the optimal mode, duration, and frequency of CT for rotator cuff tendinopathy still remains largely unclear. Consistent with the analgesic effect reported for CT in the literature, it was detected in our study that the CT protocol of applying an ice pack for 20 min five times a day for three days improved pain relief, except for pain during activity. 

Another popular intervention commonly used for pain management related to musculoskeletal problems includes KT [9,13]. There are several theories that can explain KT’s pain-relieving effect. The most believable theory is the gate control theory. It is thought that the non-painful mechanical stimulation of the skin through KT may inhibit or interfere with signals from pain fibers, thus reducing pain. It is believed that KT stimulates the neuromuscular pathway by increasing the afferent feedback. Increasing the afferent stimulation of large-diameter nerve fibers can reduce the effect of small-diameter nerve fibers that transmit pain. A KT application reduces pain by stimulating the pain relief mechanism descending from the upper centers of the brain. Another proposed mechanism is that lifting the skin with KT results in improved blood circulation and lymph flow to remove pain substances and reduces the pressure on the subcutaneous nociceptors [37,38]. Moreover, some studies have reported that the short-term positive effects of KT on pain relief may be due to a feedback mechanism of taping or a placebo effect [38,39]. Although KT has been widely used clinically, the current scientific evidence is controversial regarding its role in the treatment of shoulder pain. A systematic review and meta-analysis of 12 randomized controlled trials reported that there is insufficient evidence to support the use of KT in clinical practice as a treatment; however, there is limited evidence of its benefit as a complementary treatment for shoulder pain [39]. Another review by Gianola et al. that analyzed 23 trials revealed that the use of KT for rotator cuff disease had uncertain effects on the self-reported pain, function, pain with motion, and active range of motion when compared to sham taping or other conservative treatments, given that the certainty of the evidence was very low [40]. On the contrary, a systematic review synthesized the evidence on the effectiveness of KT in participants with painful conditions managed in primary healthcare practices. KT was found to be more effective than minimal intervention, including no taping, sham taping, and the usual care, at reducing pain in individuals with more than 4 weeks of musculoskeletal pain [41]. Simsek et al. compared the short-term effects of KT with exercise therapy to exercise therapy with sham taping in patients with shoulder impingement syndrome (SIS). It was stated that the KT group showed a statistically significant reduction in their pain level in comparison with sham taping, on both the 5th day and the 12th day [42]. Similarly, Shakeri et al. found a significant reduction in the pain intensity during movement or at night immediately after a KT application in patients with SIS [43]. Moreover, Miccinilli et al. reported that short-term KT (during two consecutive weeks) applied to patients with rotator cuff tendinopathy decreased their pain [14]. These results are consistent with our findings. A KT application for three days, with a standardized home exercise program, improved pain relief during the night, rest, and activity. All the pain scores using the NRS and SPADI were significantly reduced in the KT group. Furthermore, a statistically significant improvement was found in the pain scores, except for the resting pain, in the KT group compared with the CT group. The mean initial resting pain scores on the NRS corresponded to mild pain according to the cut-off points of pain severity (NRS resting pain scores ≤ 5), whereas the night pain and activity pain scores indicated moderate pain (NRS scores > 5 and <8) [44]. In this regard, it could be suggested that KT and CT are effective for mild pain, but KT is more effective than CT for moderate pain in patients with RCT.

RCT causes significant functional disability. Several factors, including pain, tissue injury, decreased muscle strength, and a limited ROM, can influence the overall upper extremity function in RCT. Pain and dysfunction are the most common problems reported by patients with RCT. Therefore, most of the interventions used in the treatment of RCT focus on reducing pain and increasing shoulder function [5,45]. Dupuis et al. applied an ice wrap, within a towel, on a painful shoulder for 15 min three times a day for two weeks in patients with acute rotator cuff tendinopathy and compared it with gradual reloading exercises. In this randomized study, the researchers found that short-term cryotherapy significantly improved pain relief and function, but not the shoulder ROM, in patients with acute RCT. When the results of the groups were compared, there were no significant differences between them. The researchers also reported that the mean initial ROM values were close to the normative values, so it was not surprising that the ROM did not change significantly over time [3]. Parle et al. indicated significant improvements in pain relief and function following one week of ice application in patients with acute RCT [4]. In the present study, neither the function nor the ROM improved in the CT group, although the mean initial ROM values were not close to the normative values. In contrast to previous studies, there was no improvement in pain relief during activity with the cold application in our study. We assumed that the lack of an improvement in the ROM, grip strength, and function may be attributable to this finding about pain. Subası et al. investigated the efficacy of KT, applied using the insertion–origin muscle and space-correction technique, compared to subacromial injection therapy with a three-month exercise program in patients with SIS. KT was applied three times over five consecutive days, with a two-day interval between each application. They reported that KT was found to have positive effects on the functional status, pain relief, and ROM [46]. Kaya et al. found that the DASH and VAS scores at night, at rest, and during movement significantly improved with two weeks of the KT application, including space and lymphatic correction techniques. They suggested that KT may be an alternative option for the treatment of SIS, especially when an immediate effect is needed [47]. Silva et al. examined the efficacy of KT applied according to the protocol for RC tendinitis/shoulder impingement suggested by Kase, both isolated and combined with exercise, on the pain relief and function in patients with RCT. They observed a significant improvement in pain relief, function, and the ROM in the KT group, but as expected, more expressive results were obtained when KT was combined with the exercise program. Based on these findings, they suggested that KT is an essential adjunct in the recovery of rotator cuff injuries [48]. Our positive results for the KT group, where KT was applied according to the protocol for RCT suggested by Kase [16] in terms of the function, shoulder ROM, grip strength, and pain relief, are in accordance with the results of previous studies [46,47,48]. From another point of view, it has been stated that KT may improve postural alignment by increasing cutaneous mechanoreceptor stimulation [43]. In this context, the favorable effects of KT on proprioceptive feedback may facilitate functional movement by providing a support over the joint during activity. In this study, the continuous application of KT in the shoulder region of the individuals for three days may have elicited these favorable effects. 

In our study, there were significant differences between the two groups in favor of the KT group, except for the resting pain. It is known that shoulder pain significantly impacts function [49]. In addition, Anwer et al. stated that flexion, abduction, and rotation ROMs were most significantly associated with the severity of pain and physical disability in patients with shoulder dysfunction [50]. The hand grip strength has been proposed as an indicator of rotator cuff function [51]. Considering all these relationships, it is not surprising that, in the KT group, where improvements in all the pain scores were observed, there were also favorable improvements in the function, ROM, and grip strength. The improvements in pain relief may have enhanced the ROM, the grip strength, and finally, the function. On the contrary, a lack of improvement in the ROM, grip strength, and function in the CT group may be attributable to the activity pain, in which no significant change was observed. These results may have led to KT being more effective than CT in terms of pain (except for resting pain), function, the ROM, and the grip strength. 

The positive results yielded in this study indicate that in clinical practice, a KT application with inhibition technique of supraspinatus and deltoid muscles and mechanical correction technique for three days combined with a standardized home exercise program (including pain-free shoulder ROM exercises, isometric exercises, and stretching exercises for the posterior capsule) might improve pain, function, the shoulder ROM, and the grip strength in individuals with RCT. In addition, with the previous home exercise program, a CT protocol of applying an ice pack for 20 min five times a day for three days could relieve the resting and night pain in patients with RCT. Current evidence suggests that a KT application could be a more effective therapeutic choice than a CT application for improving night pain, activity pain, and function during the short-term rehabilitation of RCT patients.

To our knowledge, this is the first randomized clinical trial comparing the effects of KT and CT on pain relief and function in individuals with RCT. However, this study has several limitations. First, the subjects were followed up for a short-term period (three days). Therefore, the present findings failed to show the sustainability of the effects of these intervention programs. Second, there was no control group that only received a standardized home exercise program. Hence, it was difficult to clearly define whether the impact on pain relief and function in RCT was due to KT or CT. Lastly, the ice application and home exercise program follow-up were performed according to the participants’ statements in the diary. For further understanding, long-term follow-up studies on the effects of KT and CT with control groups and more objective methods in monitoring cold applications are needed.

## 5. Conclusions

The present study suggests that both KT and CT, in conjunction with an exercise program, could be used as adjunctive modalities for resting and night pain relief in the short-term rehabilitation of RCT. Additionally, the findings revealed that combining a KT application with an exercise program might be an effective therapeutic choice for improving activity pain, function, the ROM, and the grip strength in individuals with RCT in the short term. This study also provides evidence that KT could be more effective than CT at reducing night and activity pain and improving upper extremity function. Further randomized controlled trials using objective intervention monitoring and including long-term follow-up are needed to investigate the effects of CT and KT in RCT management.

## Figures and Tables

**Table 1:** Demographic and initial assessments of groups.

		KT Group (*n* = 23)	CT Group (*n* = 23)	*p*-Value	95% CI of the Difference
Age (years)		44.00 ± 5.64	44.47 ± 7.16	0.803	(−4.31, 3.35)
BMI (kg/m^2^)		26.96 ± 5.01	27.89 ± 4.38	0.505	(−3.73, 1.86)
Gender					
Female (n)		14 (60.9%)	10 (43.5%)	0.238	
Male (n)		9 (39.1%)	13 (56.5%)		
Dominance					
Right-handed (n)		19 (82.6%)	21 (91.3%)	0.381	
Left-handed (n)		4 (17.4%)	2 (8.7%)		
Affected shoulder					
Right (n)		13 (56.5%)	10 (43.5%)	0.376	
Left (n)		10 (43.5%)	13 (56.5%)		
NRS score	Resting pain	3.52 ± 2.17	4.17 ± 2.34	0.333	(−1.99, 0.69)
	Night pain	5.69 ± 2.96	5.60 ± 2.74	0.918	(−1.60, 1.78)
	Activity pain	7.39 ± 2.40	6.52 ± 2.39	0.226	(−0.55, 2.29)
DASH questionnaire score		54.77 ± 18.64	49.75 ± 15.79	0.330	(−5.24, 15.28)
SPADI score	Pain	73.04 ± 18.83	67.04 ± 17.95	0.275	(−4.93, 16.93)
	Disability	61.41 ± 20.83	52.93 ± 21.90	0.186	(−4.22, 21.18)
	Total	65.88 ± 19.24	58.35 ± 19.61	0.196	(−4.02, 19.07)
ROM (°)	Flexion	132.60 ± 43.08	137.21 ± 37.08	0.699	(−28.49, 19.28)
	Abduction	101.73 ± 45.29	101.21 ± 36.57	0.966	(−23.94, 24.98)
	İnternal rotation	54.34 ± 19.26	51.73 ± 21.77	0.669	(−9.60, 14.82)
	External rotation	53.69 ± 24.08	51.17 ± 22.04	0.715	(−11.30, 16.34)
Hand grip strength (kg)		21.99 ± 10.76	25.66 ± 13.08	0.311	(−10.88, 3.55)

Data are presented as mean ± standard deviation or n (%). KT: Kinesio taping; CT: cold therapy; BMI: body mass index; NRS: numerical rating scale; DASH: disability of the arm, shoulder, and hand; SPADI: shoulder pain and disability index; ROM: range of motion; CI: confidence interval.

**Table 2:** Intra- and inter-group comparisons of initial and final assessment scores.

						Time × Group Interaction		
			Initial Assessment (Mean ± ss)	Final Assessment (Mean ± ss)	p¹	F	p^2^	η^2^
NRS score	Resting pain	KT group	3.52 ± 2.17	2.00 ± 1.31	**<0.001**	0.163	0.688	0.004
		CT group	4.17 ± 2.34	2.86 ± 2.13	**0.001**			
	Night pain	KT group	5.69 ± 2.96	2.86 ± 2.43	**<0.001**	4.839	**0.033**	0.099
		CT group	5.60 ± 2.74	4.26 ± 2.33	**0.007**			
	Activity pain	KT group	7.39 ± 2.40	5.47 ± 2.19	**<0.001**	12.521	**0.001**	0.222
		CT group	6.52 ± 2.39	6.60 ± 2.21	0.829			
DASH questionnaire score		KT group	54.77 ± 18.64	42.66 ± 19.96	**<0.001**	12.976	**0.001**	0.228
		CT group	49.75 ± 15.79	48.77 ± 18.22	0.658			
SPADI score	Pain	KT group	73.04 ± 18.83	53.04 ± 21.17	**<0.001**	18.758	**<0.001**	0.299
		CT group	67.04 ± 17.95	62.78 ± 20.45	**0.104**			
	Disability	KT group	61.41 ± 20.83	47.28 ± 19.39	**<0.001**	18.092	**<0.001**	0.291
		CT group	52.93 ± 21.90	53.20 ± 21.69	0.910			
	Total	KT group	65.88 ± 19.24	50.32 ± 18.72	**<0.001**	24.174	**<0.001**	0.355
		CT group	58.35 ± 19.61	56.91 ± 19.90	0.482			
ROM (°)	Flexion	KT group	132.60 ± 43.08	142.17 ± 40.24	**<0.001**	15.270	**<0.001**	0.258
		CT group	137.21 ± 37.08	135.73 ± 39.54	0.463			
	Abduction	KT group	101.73 ± 45.29	113.47 ± 42.16	**<0.001**	14.329	**<0.001**	0.246
		CT group	101.21 ± 36.57	101.21 ± 38.01	1.000			
	İnternal rotation	KT group	54.34 ± 19.26	61.00 ± 19.33	**<0.001**	10.946	**0.002**	0.199
		CT group	51.73 ± 21.77	51.08 ± 22.51	0.678			
	External rotation	KT group	53.69 ± 24.08	60.95 ± 23.21	**<0.001**	9.355	**0.004**	0.175
		CT group	51.17 ± 22.04	51.21 ± 22.91	0.979			
Hand grip strength (kg)		KT group	21.99 ± 10.76	24.14 ± 11.31	**<0.001**	6.538	**0.014**	0.132
		CT group	25.66 ± 13.08	26.24 ± 12.94	0.182			

KT: Kinesio taping; CT: cold therapy; NRS: numerical rating scale; DASH: disability of the arm, shoulder, and hand; SPADI: shoulder pain and disability index; ROM: range of motion. p^1^: *p*-value for within-group comparison; p^2^: *p*-value for time × group interaction analysis of mixed-model ANOVA; η^2^: partial-eta squared.

## Data Availability

The datasets used and/or analyzed during the current study are available from the corresponding author upon reasonable request.

## Supplementary Materials

The following supporting information can be downloaded at: https://www.mdpi.com/article/10.3390/medicina60081188/s1, Figure S1: Box plots of change in NRS scores of Kinesio taping (KT) and cold therapy (CT) groups from initial assessment to day 3; Figure S2: Box plots of change in SPADI scores of Kinesio taping (KT) and cold therapy (CT) groups from initial assessment to day 3; Figure S3: Box plots of change in DASH questionnaire scores and hand grip strength values of Kinesio taping (KT) and cold therapy (CT) groups from initial assessment to day 3; Figure S4: Box plots of change in shoulder range of motion values of Kinesio taping (KT) and cold therapy (CT) groups from initial assessment to day 3.
