# Supplementary material for: Short-Term Effects of Cold Therapy and Kinesio Taping on Pain Relief and Upper Extremity Functionality in Individuals with Rotator Cuff Tendonitis: A Randomized Study
Source: Medicina (Kaunas). 2024 Jul 23;60(8):1188. doi: 10.3390/medicina60081188 (PMC11356310; doi:10.3390/medicina60081188)
Supplement: Supplementary file 1 [file medicina-60-01188-s001.zip › medicina-3071584-supplementary.pdf]

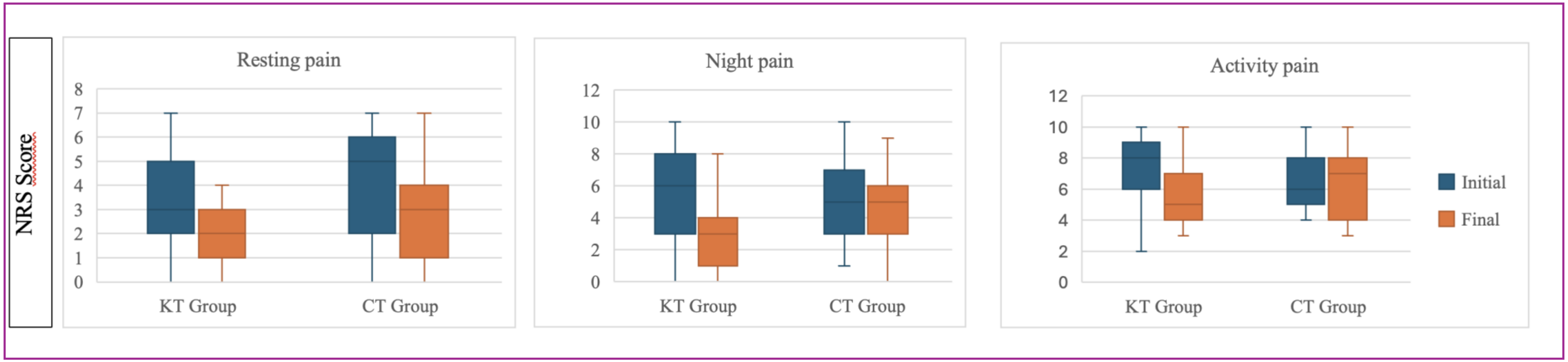

Figure S1: Box plots of change in NRS scores of kinesio taping (KT) and cold therapy (CT) groups from initial to day 3.

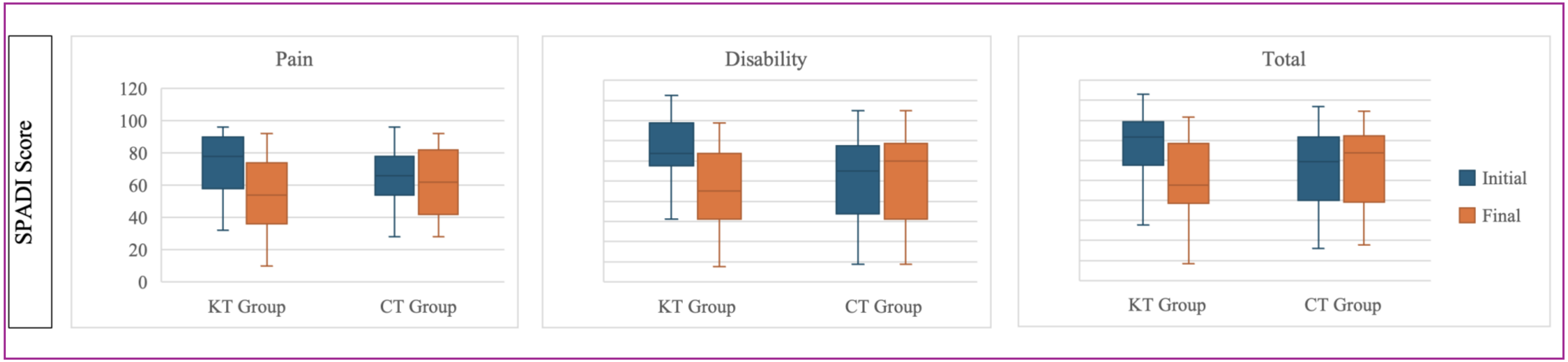

Figure S2: Box plots of change in SPADI scores of kinesio taping (KT) and cold therapy (CT) groups from initial to day 3.

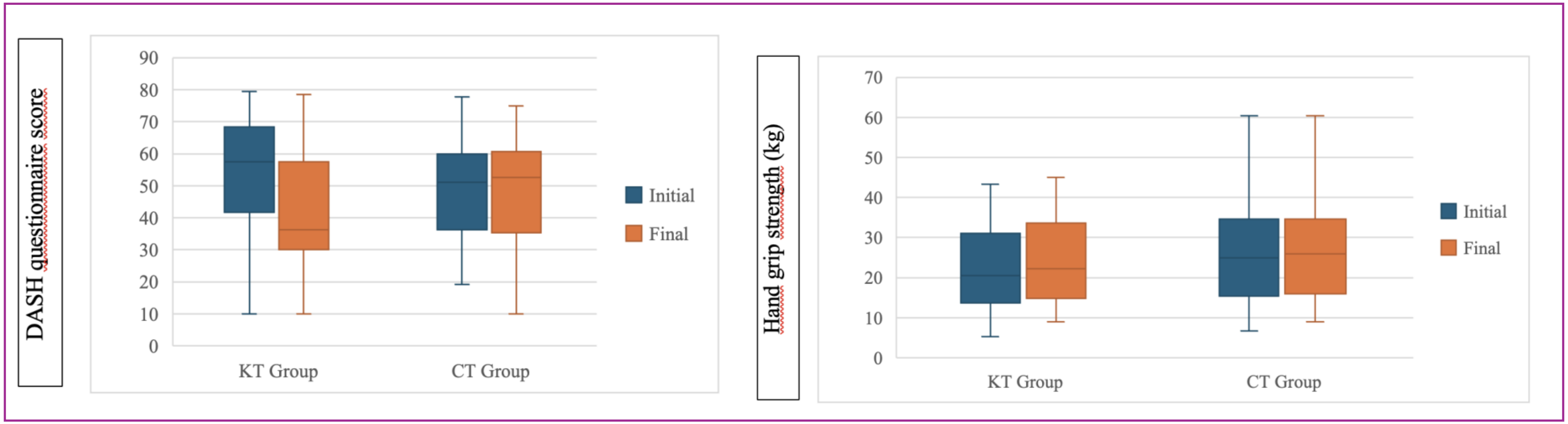

Figure S3: Box plots of change in DASH questionnaire scores and hand grip strength values of kinesio taping (KT) and cold therapy (CT) groups from initial to day 3.

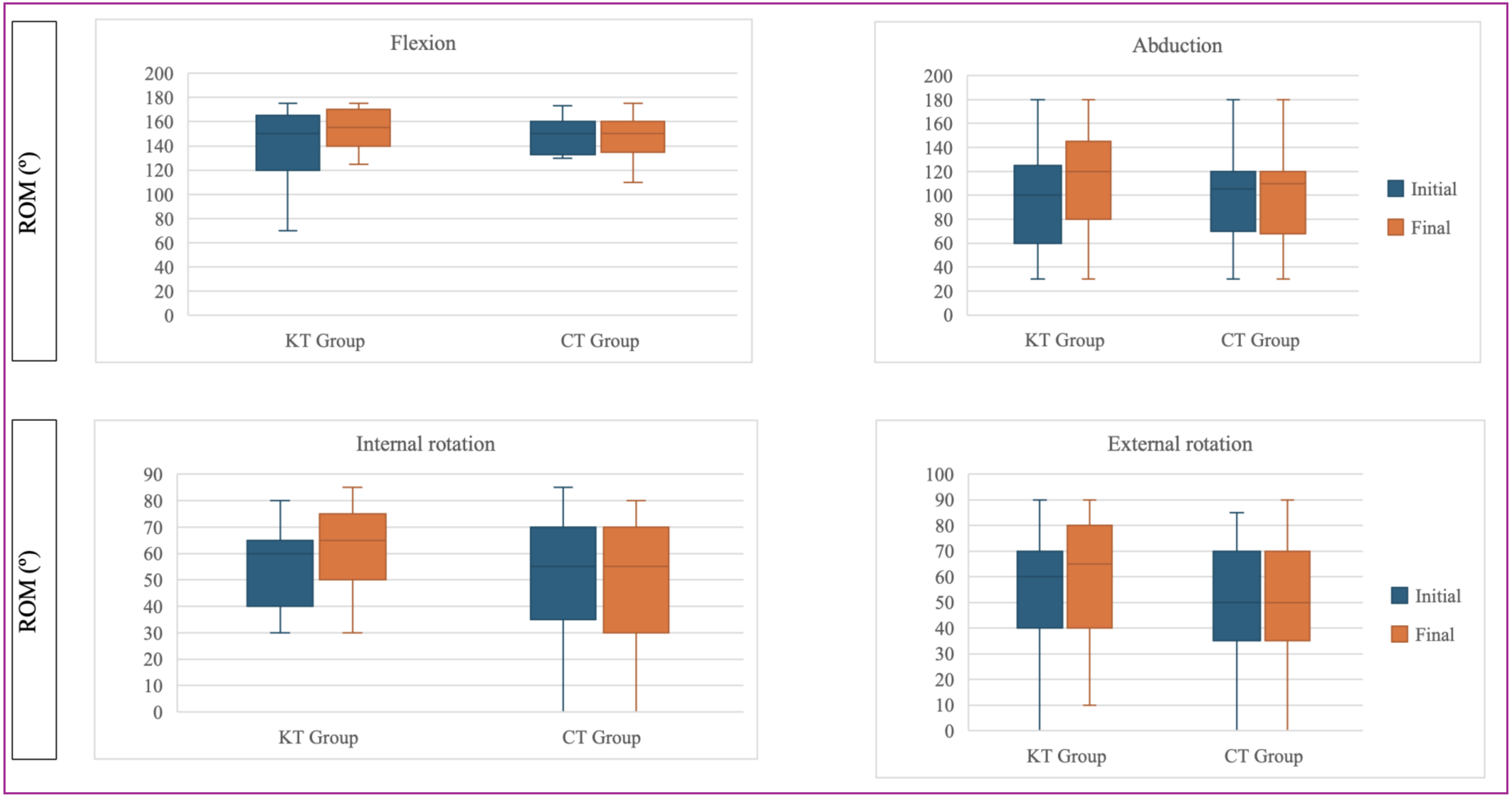

Figure S4: Box plots of change in shoulder range of motion values of kinesio taping (KT) and cold therapy (CT) groups from initial to day 3.
